# Editorial: Transfusions in the neonatal period

**DOI:** 10.3389/fped.2022.982918

**Published:** 2022-07-25

**Authors:** Merih Cetinkaya, Begum Atasay

**Affiliations:** ^1^Division of Neonatology, Department of Pediatrics, Cam Sakura City Hospital, Health Sciences University, Istanbul, Turkey; ^2^Division of Neonatology, Department of Pediatrics, Ankara University School of Medicine, Ankara, Turkey

**Keywords:** transfusion, neonatal, packed red blood cell, platelet, intravenous immune globulin (IVIG)

## Introduction

Anemia, thrombocytopenia and bleeding problems occur more frequently in sick neonates, especially in preterm infants. Therefore; packed red blood cell (PRBC), platelet and fresh frozen plasma (FFP) transfusions have been widely used for the management of these infants during the hospitalization period in neonatal intensive care unit (NICU). Approximately 40% of low birth weight infants and 90% of extremely low birth weight infants were reported to require red blood cell transfusion in NICU. However, there are significant and large worldwide variations in clinical practice in NICUs due to limited number of evidence-based guidelines and standardized protocols (1–5). Application of evidence-based guidelines will be helpful to perform universal approach to neonatal transfusions as well as to minimize the adverse effects of transfusion and wastage of products. Therefore, further clinical studies are required in the field of neonatal transfusions.

The aim of the special issue of Frontiers in Pediatrics Research Topic “*Transfusion in the Neonatal Period*” was to collect articles about all aspects of neonatal transfusion that might be helpful for the healthcare staff to develop new guidelines and insights. This special issue includes one mini review and three original studies.

## Packed red blood cell transfusion

The main purpose of PRBC transfusions in neonates is to optimize oxygen delivery to tissues. Although transfusion can save lives in many conditions, neonatal PRBC transfusions were reported to be associated with several prematurity-related morbidities including necrotizing enterocolitis (NEC), bronchopulmonary dysplasia (BPD), retinopathy of prematurity (ROP), intraventricular hemorrhage (IVH) and long-term abnormal neurodevelopment in preterm infants (1). However, two large prospective trials comparing restrictive or liberal transfusion approaches in preterm infants did not show any negative effects of PRBC transfusions on neonatal morbidities and mortality (6, 7). Although conflicting data about the effect of transfusion on development of NEC continues, several studies reported higher risk of ROP in preterm infants exposed to PRBC transfusions (1). Increased HbA content of PRBC suspensions, decreased HbF levels that result with greater oxygen release to tissues after transfusion, higher risk of endothelial injury due to release of free oxygen radicals, release of mediators and endothelial cell activation were all reported as the possible mechanisms associated with ROP development after PRBC transfusions. The number (≥2) and time (during the first 10 days of life) of PRBC transfusions were found to be associated with severe ROP in very low birth weight (VLBW) infants (8, 9). A recent meta-analysis also reported RBC transfusion as an independent risk factor for the development of ROP, especially in younger preterm infants (10). In the light of these findings, Teofili et al. investigated the possible effect of postmenstrual age (PMA) of preterm neonates at transfusion on ROP risk and found that preterm infants receiving more than one PRBC transfusion before 29 weeks of PMA might be exposed to a greater disturbance of retinal vascularization. Therefore, the authors recommended developing strategies that avoid transfusion at such PMA for reducing the risk of severe ROP in preterm infants.

In addition to the aforementioned morbidities, PRBC transfusion has been associated with metabolic complications such as hypoglycemia, hyperkalemia, and hypocalcemia in neonates. The risk of hyperkalemia was reported to be related with the amount of K^+^ load delivered with RBC transfusion. Irradiation was also suggested to cause hyperkalemia by damaging the membranes (11). Hyperkalemia due to transfusion may also result in transfusion-associated hyperkalemic cardiac arrest (TAHCA) in neonates, infants and children. As the incidence of TAHCA in neonates and children are not known precisely, it will be important for both neonatologists and pediatricians to be aware of this rare but important complication. The rapid neonatal transfusions, dated PRBC usage, off-site irradiation, prolonged storage and irradiation times, and use of central lines for transfusion may all increase the risk of hyperkalemia and cardiac arrest in infants. Therefore, it is important to know the issues associated with blood storage, the red cell storage lesions, and variables that increase extracellular [K^+^] levels which result in hyperkalemia and possible cardiac arrest and TAHCA. Hence, we believe that the review by Burke et al. in this special issue will provide important data about hyperkalemia, cardiac arrest and TAHCA in newborns, infants and children.

## Platelet transfusion

Thrombocytopenia is defined as a platelet blood count of less than 150,000/μL. The decreased production of platelets, increased platelet consumption, increased extravascular loss, or a combination of these factors were all suggested as the possible mechanisms of neonatal thrombocytopenia (12, 13). Among all causes, bleeding in association with severe thrombocytopenia is the most common indication for platelet transfusion. Platelet transfusions are usually performed in order to prevent or treat active bleeding (intraventricular, lung or gastrointestinal) in high risk infants. Nowadays, approximately 80% of platelet transfusions have been performed prophylactically. There is also limited number of guidelines using different platelet thresholds for platelet transfusions in neonates (12, 13). Therefore, it is important to determine new markers that may be used for the decision of platelet transfusion in neonates. Immature platelet count (IPC) was suggested as a novel marker for the assessment of neonatal thrombocytopenia (13). The immature platelets are newly released platelets that are <24 h old. They are larger and hemostatically more active than “older” platelets. Determination of IPC was reported to be useful for the management of platelet transfusion in neonates (14). Sallmon et al. evaluated the possible relationship between IPC and thrombopoietin (Tpo) concentrations in thrombocytopenic and non-thrombocytopenic VLBW infants and also healthy term infants in a clinical study. Herein, the authors found similar developmental changes in all study groups and recommended that future studies should be performed that consider serial ICP evaluation for the decision of platelet transfusion in neonates.

## Intravenous immunoglobulin transfusion

Intravenous immunoglobulin (IVIG) therapy is defined as the use of a combination of antibodies obtained from healthy human donors for treatment of several diseases. IVIG has been proposed as a potential therapy to treat hemolytic disease of the newborn (HDN) by decreasing the severity of hemolysis and hyperbilirubinemia in infants (15). IVIG has been suggested to block the antibodies' receptors located on the red blood cells' surface, prevent the antigen/antibody interactions, decrease recognition of the targeted red blood cells and subsequently reduce the degree of hemolysis (15). However, the role of IVIG for the treatment of hemolytic disease due to ABO incompatibility is controversial and there is not enough evidence for routine IVIG therapy in HDN. Okulu et al. investigated the role of IVIG in the prevention of exchange transfusion in infants with ABO HDN who admitted with bilirubin levels at or above the level of exchange transfusion in a multicenter study. They showed that IVIG therapy did not prevent an exchange transfusion nor decrease the duration of phototherapy in this large cohort of infants with HDN due to ABO incompatibility.

## Conclusion

The collection of articles on neonatal transfusions in this Frontiers Topic highlights our current understanding, knowledge gaps and approaches for all types of Transfusions in the neonatal period. We also believe that these articles may describe priorities for future research on this topic.

## Author contributions

MC and BA contributed to the editorial and agreed on its final version. Both authors contributed to the article and approved the submitted version.

## Conflict of interest

The authors declare that the research was conducted in the absence of any commercial or financial relationships that could be construed as a potential conflict of interest.

## Publisher's note

All claims expressed in this article are solely those of the authors and do not necessarily represent those of their affiliated organizations, or those of the publisher, the editors and the reviewers. Any product that may be evaluated in this article, or claim that may be made by its manufacturer, is not guaranteed or endorsed by the publisher.

## References

[1] Goel R Josephson CD. Recent advances in transfusions in neonates/infants. Version 1. F1000Res. (2018) 7:F1000. 10.12688/f1000research.13979.1 29904575 PMC5964626

[2] Villeneuve A Arsenault V Lacroix J Tucci M. Neonatal red blood cell transfusion. Vox Sang. (2021) 116:366–78. 10.1111/vox.13036 33245826

[3] Keir A Grace A Stanworth S. Closing the evidence to practice gap in neonatal transfusion medicine. Semin Fetal Neonatal Med. (2021) 26:101197. 10.1016/j.siny.2021.101197 33541808

[4] Cetinkaya M Atasay B Perk Y. Turkish neonatal society guideline on the transfusion principles in newborns. Turk Pediatri Ars. (2018) 53:S101–8. 10.5152/TurkPediatriArs.2018.01810 31236023 PMC6568302

[5] New HV Berryman J Bolton-Maggs PH Cantwell C Chalmers EA Davies T. Guidelines on transfusion for fetuses, neonates and older children. Br J Haematol. (2016) 175:784–828. 10.1111/bjh.14233 27861734

[6] Franz AR Engel C Bassler D Rüdiger M Thome UH Maier RF. Effects of liberal vs restrictive transfusion thresholds on survival and neurocognitive outcomes in extremely low-birth-weight infants: the ETTNO randomized clinical trial. JAMA. (2020) 324:560–70. 10.1001/jama.2020.10690 32780138 PMC7420159

[7] Kirpalani H Bell EF Hintz SR Tan S Schmidt B Chaudhary AS. Higher or lower hemoglobin transfusion thresholds for preterm infants. N Engl J Med. (2020) 383:2639–51. 10.1056/NEJMoa2020248 33382931 PMC8487591

[8] Bas AY Demirel N Koc E Isik DU Hirfanoglu IM Tunc T. Incidence, risk factors and severity of retinopathy of prematurity in Turkey (TR-ROP study): a prospective, multicentre study in 69 neonatal intensive care units. Br J Ophthalmol. (2018) 102:1711–6. 10.1136/bjophthalmol-2017-311789 29519879 PMC6287567

[9] Lust C Vesoulis Z Jackups R Liao S Rao R Mathur AM. Early red cell transfusion is associated with development of severe retinopathy of prematurity. J Perinatol. (2019) 39:393–400. 10.1038/s41372-018-0274-9 30459388 PMC6391181

[10] Zhu Z Hua X Yu Y Zhu P Hong K Ke Y. Effect of red blood cell transfusion on the development of retinopathy of prematurity: a systematic review and meta-analysis. PLoS ONE. (2020) 15:e0234266. 10.1371/journal.pone.0234266 32512582 PMC7279893

[11] Kim DH. Transfusion practice in neonates. Korean J Pediatr. (2018) 61:265–70. 10.3345/kjp.2018.06849 30185018 PMC6172519

[12] Patel RM Josephson CD. Neonatal and pediatric platelet transfusions: current concepts and controversies. Curr Opin Hematol. (2019) 26:466–72. 10.1097/MOH.0000000000000542 31503020 PMC6935356

[13] Ree IMC Lopriore E. Updates in neonatal hematology: causes, risk factors, and management of anemia and thrombocytopenia. Hematol Oncol Clin North Am. (2019) 33:521–32. 10.1016/j.hoc.2019.01.013 31030817

[14] MacQueen BC Christensen RD Henry E Romrell AM Pysher TJ Bennett ST. The immature platelet fraction: creating neonatal reference intervals and using these to categorize neonatal thrombocytopenias. J Perinatol. (2017) 37:834–8. 10.1038/jp.2017.48 28383532 PMC6192246

[15] Alsaleem M. Intravenous immune globulin uses in the fetus and neonate: a review. Antibodies. (2020) 9:60. 10.3390/antib9040060 33158209 PMC7709108

